# Starchy and fibrous feedstuffs differ in their in vitro digestibility and fermentation characteristics and differently modulate gut microbiota of swine

**DOI:** 10.1186/s40104-022-00699-y

**Published:** 2022-05-03

**Authors:** Utsav P. Tiwari, Rabindra K. Mandal, Kabi Raj Neupane, Birendra Mishra, Rajesh Jha

**Affiliations:** 1 Department of Human Nutrition, Food and Animal Sciences, University of Hawaii at Manoa, 1955 East-West Rd, Honolulu, HI 96822 USA; 2 Indiana University School of Medicine, Indiana, USA; 3 Math and Sciences Division, Leeward community college, Pearl City, HI 96782 USA

**Keywords:** Fermentation, Fiber, Gut health, Microbial diversity, Starch, Swine

## Abstract

**Background:**

Alternative feedstuffs may contribute to reducing feed costs of pig production. But these feedstuffs are typically rich in fiber and resistant starch (RS). Dietary fibers and RS are fermented in the gastrointestinal tract (GIT) and modulate the microbial community. Certain microbes in the GIT can promote host health, depending on the type of fermentation substrates available. In this study, six alternative feedstuffs (three starchy: Okinawan sweet potato, OSP; yam, and taro, and three fibrous: wheat millrun, WMR; barley brewers grain, BBG; and macadamia nut cake, MNC) were evaluated for their in vitro digestibility and fermentation characteristics and their effects on pig’s hindgut microbial profile. After 2 steps of enzymatic digestion assay, residues were fermented using fresh pig feces as microbial inoculum, and gas production was recorded periodically for 72 h and modeled for fermentation kinetics. After fermentation, the residual liquid phase was analyzed for short-chain fatty acid (SCFA), and the solid phase was used to determine the nutrient’s digestibility and microbial community.

**Results:**

In vitro ileal digestibility of dry matter and gross energy was higher in starchy than fibrous feedstuffs. Total gas and SCFA production were significantly higher (*P* < 0.001) in starchy feedstuffs than fibrous feedstuffs. Both acetate and propionate production was significantly higher (*P* < 0.001) in all starchy feedstuffs than BBG and MNC; WMR was in between. Overall alpha diversity was not significantly different within and between starchy and fibrous feedstuffs. Beta diversity (measured using bray Curtis dissimilarity distance) of starchy feedstuffs was significantly different (*P* < 0.005) than fibrous feedstuffs.

**Conclusion:**

Starchy feedstuffs acted as a substrate to similar types of microbes, whereas fibrous feedstuffs resulted in a more diverse microbial population. Such alternative feedstuffs may exert comparable beneficial effects, thus may be included in swine diets to improve gut health.

## Introduction

The high feed cost for swine is one of the major factors affecting the net profit of the swine production. To facilitate increasing production at a reasonable cost while maintaining the health of swine, new feed sources must be investigated. Most of the cereal grains and co-products used in swine diets contain a variable amount of fermentable carbohydrates in the form of resistant starch (RS) and nonstarch polysaccharides (NSP) [1]. This RS and NSP resist the digestion in the stomach and enzymatic hydrolysis in the small intestine. Hence, the undigested substrates in the small intestine are available for fermentation in the large intestine, resulting in the production of short-chain fatty acids (SCFA) [2], which are absorbed in the pig intestine and serves as a source of energy [2]. The SCFA, such as butyrate, may have a impact on pig health as butyrate has been shown to improve intestinal functionality [2, 3] by providing energy to enterocytes and maintaining gut barrier integrity [2]. The SCFA production is directly proportional to the amount of substrate fermented [1]. However, the fermentation characteristics of fibers and starches vary based on their physicochemical characteristics. Thus, manipulating the substrate that reaches the large intestine can influence the composition of SCFA produced. There are varieties of starchy and fibrous feedstuffs (like Okinawan sweet potato, OSP; yam, taro, wheat millrun, WMR; barley brewers grain, BBG; macadamia nut cake, MNC) available in Hawaii and elsewhere that have the potential to be used in swine diets due to their reasonable nutrient profile and energy digestibility [4]. However, little is known about the fermentation characteristics of these feedstuffs and their effect on the microbial community in the large intestine of swine.

The link between gut microbes and health has been well documented over the years and continues to be an important topic of investigation. Establishing the link between feedstuffs and gut microflora is vital to understanding how various feedstuffs affect the microbial community in the gut, thereby, gut health and performance of swine. The composition of the gut microbiota is intimately connected to the health of the host. Studies have shown that diet impacts the microbial community in the gastrointestinal tract (GIT) [5]. Therefore, it is important to evaluate the effects of feed on the microbial population [6].

The effects of different potential feed on gut microbial populations need to be examined that would help to formulate their inclusion level in the swine diet. Bacterial diversity in ecosystems cannot be adequately represented by traditional culturing methods [7]. Therefore, 16S rDNA sequencing-based methods can be used to understand what populations are impacted by factors, such as diet changes. Furthermore, the structure of ribosomes consists of highly conserved and variable regions, making it an ideal choice for identifying bacterial species [7, 8]. Hence, it is important to make a dietary strategy that can consistently increase SCFA production throughout the colon by promoting gut microbiomes without compromising an animal’s growth performance and health. The objective of this study was to examine the difference in fermentation characteristics of starchy and fibrous feedstuffs, and their effect on the microbial profile in the large intestine of swine.

## Materials and methods

### Feedstuffs

Three starchy feedstuffs (OSP, yam, and taro) and three fibrous feedstuffs (WMR, BBG, and MNC) were used in this study. These feedstuffs were selected based on their nutrient profile and potential to be used in the swine diets [4, 9].

### In vitro enzymatic digestion

The 2-step in vitro digestion technique [10] simulates the digestion activities occurring in the upper gastrointestinal tract of the pig and provides information on the apparent ileal digestibility of dry matter (DM), gross energy (GE), and other nutrients.

All the six feed samples were ground to pass through a 1.0-mm mesh screen and subjected to 2-step in vitro digestion as described by Boisen and Fernandez [10]. Briefly, 2 g sample was weighed in a conical flask. A phosphate buffer solution (100 mL, 0.1 mol/L, pH 6.0) and an HCl solution (40 mL, 0.2 mol/L) was poured into the flasks. Two mL of chloramphenicol (Sigma C-0378, Sigma-Aldrich Corp., St. Louis, MO, USA) solution (0.5 g/100 mL ethanol) was added to prevent bacterial growth during hydrolysis. Fresh pepsin solution (4 mL, 20 g/L porcine pepsin, Sigma P-0609) was added, and the flasks were placed in a water bath at 39 °C for 2 h under gentle agitation (50 r/min). Afterward, 40 mL phosphate buffer (0.2 mol/L, pH 6.8) and 20 mL of 0.6 mol/L NaOH were added. Fresh pancreatin solution (12 mL, 100 g/L pancreatin; Sigma P-1750) was added, and hydrolysis was continued for 4 h under the same conditions. After hydrolysis, the residues were collected by filtration on a nylon cloth (42 μm), washed with ethanol (2 × 25 mL 95% ethanol) and acetone (2 × 25 mL 99.5% acetone), dried for 12 h at 60 °C and weighed. The enzymatic hydrolysis was repeated 3 times to obtain enough samples for the in vitro fermentation and their analysis. Hydrolyzed residues from the different replicates and batches of the same ingredients were pooled for subsequent analyses (DM and GE) and in vitro fermentation.

### In vitro microbial fermentation

The in vitro fermentation technique simulates the microbial fermentation occurring in the large intestine of the swine [11]. It provides information on the total gas and fermentation metabolites produced by microbial fermentation, which are directly proportional to the amount of substrate fermented.

The fermentation rate of the hydrolyzed substrates was assessed in vitro, using a cumulative gas-production technique adapted to the pig as previously described [11]. Briefly, 200 mg samples were incubated at 39 °C (in a shaking water bath with 50 r/min) in a 125-mL glass bottle with 30 mL buffer solution containing macro- and micro-minerals and a fecal inoculum. Three growing swine from a local commercial farm herd fed a standard commercial diet devoid of antibiotics were used as donors for the fecal inoculums. The inoculum prepared from feces was diluted 20 times in the buffer solution, filtered through a 250-μm screen, and transferred into the bottle with fermentation substrates. Bottles were sealed with a rubber stopper and placed for incubation. An anaerobic environment was maintained throughout the experiment, from inoculum preparation until incubation, by flushing with CO_2_ gas. The gas generated by fermentation and CO_2_ released by buffering of SCFA produced during the fermentation was measured at 0, 2, 5, 8, 12, 18, 24, 36, 48, and 72 h using a pressure transducer (GP:50 SIN-54978, Grand Island, NY, USA), fitted with digital data tracker (Tracker 211, Intertechnology Inc., Don Mills, ON, Canada). The bottles were vented after every measurement. Fermentation was stopped at 72 h of incubation by quenching the bottles in ice water, and samples were collected from the bottles and stored frozen for SCFA analysis. Also, 5 mL of homogenous mix solution liquid after fermentation was collected for microbial analysis and processed as described below.

The experimental scheme for in vitro fermentation study was as follows: 6 samples × 6 replicates + 6 blanks (containing the only inoculum) repeated over 3 runs (batches).

### Nutrient analysis

The feedstuffs were ground to pass through a 1.0-mm mesh screen using a laboratory mill. Ground samples were subjected to proximate analysis according to the Association of Official Analytical Chemists standard procedures [12] with specific methods as follows: DM (135 °C for 2 h, AOAC 930.15), ash (AOAC 942.05), CP by determining N using Kjeldahl method (AOAC 976.05, CP = N × 6.25), ether extract (AOAC 920.39; using Soxhlet apparatus and petroleum ether), ADF (AOAC 973.18), and NDF (AOAC 2002.04). Total starch content was determined using a commercial test kit (Megazyme International, Wicklow, Ireland). The GE content was determined using an oxygen bomb calorimeter (Parr Bomb Calorimeter 6200, Parr Instrument Co., Moline, IL, USA). Total and soluble NSP of fibrous feedstuffs with their constituent sugars were quantified by gas chromatography (GC) following procedures and calculations as previously described [13]. Chromatographic analysis was done using a GC system (TRACE™ 1300 gas chromatograph; Thermo Scientific, Waltham, MA, USA) equipped with a flame ionization detector and a fused silica capillary column (DB-17HT; Agilent Technologies, Wilmington, DE, USA), using 2-deoxy-D-glucose as an internal standard.

The residue after in vitro digestion was analyzed for DM (method 930.15) and GE (method 984.13A-D) using the standard procedure [12] and used to calculate the apparent in vitro digestibility.

### Short-chain fatty acid analysis

Samples collected from the bottles at the end of fermentation were centrifuged, and supernatant from each bottle was analyzed for SCFA. Concentrations of SCFA in post-fermentation solution were determined using a GC system. Briefly, 0.8 mL of the test sample (supernatant from centrifuged at 2500×*g *for 10 min at 4 °C) were added in a tube with 0.2 mL of 25% phosphoric acid and 0.2 mL of internal standard solution (150 mg of 4-methyl-valeric acid, S381810, Sigma-Aldrich) and vortexed thoroughly. Samples were analyzed for SCFA (i.e., acetate, propionate, butyrate, isobutyrate, valerate, isovalerate, and caproate) using a GC system (TRACE™ 1300 gas chromatograph; Thermo Scientific, Waltham, MA, USA) with a Stabilwax-DA column (30-m × 0.25-mm internal diameter; Restek, Bellefonte, PA, USA). A flame-ionization detector was used with an injector temperature of 170 °C and a detector temperature of 190 °C. Branched-chain fatty acids (BCFA) content was calculated as the sum of iso-butyrate and iso-valerate.

### DNA extraction and metagenomic analysis

After the centrifugation of the fermentation product, the pellet residue from 9 replicates of each treatment from 3 batches were pooledin two tubes to make two replicates. Those two replicates of each treatment were used to extract genomic DNA using a Repeated Bead Beating Plus Column Method (RBB + C) with the QIAamp DNA Stool Mini Kit. Purified genomic DNA was isolated by removing the RNA and proteins using QIAamp Mini spin columns. Extracted DNA was then quantified using a GE NanoVue spectrophotometer, followed by examining its quality in a 0.8% (w/v) agarose gel. Metagenomic analysis of the 16S rRNA V3 and V4 regions was conducted using the Illumina MiSeq system in the John A. Burns School of Medicine, University of Hawaii at Manoa. The following primers were used before sequencing (in standard IUPAC nucleotide nomenclature): 16S Amplicon PCR Forward Primer = 5´-TCGTCGGCAGCGTCAGATGTGTATAAGAGACAGCCTACGGGNGGCWGCAG-3´.

16S Amplicon PCR Reverse Primer = 5′-

GTCTCGTGGGCTCGGAGATGTGTATAAGAGACAGGACTACHVGGGTATCTAATCC-3´.

For further processing, Quantitative Insights Into Microbial Ecology (QIIME™ version 2.0 release 2019.4) was used to import demultiplexed paired-end reads of 300 bp in length for all samples. After importing into QIIME2, the DADA2 pipeline was used to denoise, trim, and filter these paired-end sequences. The filtered sequences were subjected to align-to-tree-mafft-fasttree pipeline from the QIIME phylogeny plugin to generate an unrooted and rooted tree for phylogeny. A Naïve Bayes classifier pre-trained on the Greengenes 13.99% OTU was used for taxonomy analysis. The diversity plugin method named core-metrics-phylogenetic was used to conduct alpha and beta diversity analysis on sampling depth of 10,000 frequency. Alpha diversity results were presented as Shannon Index and observed OTUs, while Bray Curtis metrics and unweighted UniFrac were applied for beta diversity.

### Statistical analysis

The in vitro dry matter and gross energy digestibility, total gas production, and SCFA production were compared among treatments using the MIXED procedure of SAS 9.2 software (SAS Institute Inc., Cary, NC, USA). Feedstuffs were the fixed factor in the model, and subsample (during both in vitro digestion and fermentation) and batch (only during in vitro digestion) as random factors. Means were separated by the Tukey method using the “pdmix” macro of SAS, and differences among variables were declared significant at a probability level of 0.05.

Statistical analysis of differentially abundant bacteria between fiber and starch-based feeds at the genus level was performed using a linear discriminant analysis (LDA) effect size (LEfSe) method.

## Results

The nutrient profile of starchy and fibrous feedstuffs is presented in Table 1.

**Table 1:** Nutrient profile of starchy and fibrous feedstuffs, % DM basis [4, 9]

Variable	OSP^1^	Yam	Taro	WMR^2^	BBG^3^	MNC^4^
Dry matter	94.80	94.50	97.70	96.70	97.10	91.20
Ash	2.00	2.80	2.40	1.80	8.70	3.70
Crude protein	4.80	5.30	8.80	11.80	15.90	25.50
Ether extract	2.80	2.00	1.90	4.10	1.80	11.90
Acide detergent fiber	5.70	8.10	10.40	24.20	34.10	28.00
Neutral detergent fiber	8.00	9.70	11.50	35.00	42.10	35.80
Hemi-cellulose	2.30	1.50	1.10	10.80	8.00	7.80
Gross energy, Kcal/kg	4134	4154	4333	4794	4270	5581

^1^Okinawan sweet potato

^2^Wheat millrun

^3^Barley brewers grain

^4^Macadamia nut cake

The NSP concentration of different fibrous feedstuffs is presented in Table 2. The highest concentration of NSP, as well as cellulose, was found in BBG. A higher concentration of soluble NSP was present in WMR. Arabinose substitution on xylan backbone (A:X ratio) was higher in MNC.

**Table 2:** The nonstarch polysaccharide content of fibrous feedstuffs, % DM basis [4, 9]

NSP	WMR^1^	BBG^2^	MNC^3^
NSP			
Total	29.00	38.80	36.40
Soluble	11.82	4.80	5.60
Cellulose	6.54	12.34	7.20
Glucose			
Total	9.28	4.80	5.26
Soluble	7.20	2.46	2.46
Xylose			
Total	6.46	12.16	12.84
Soluble	1.07	1.05	1.68
Arabinose			
Total	3.68	8.02	9.12
Soluble	2.32	1.01	1.01
Galactose			
Total	2.06	0.77	1.05
Soluble	0.67	0.175	0.27
Mannose			
Total	1.02	0.74	0.90
Soluble	0.34	0.11	0.74
A:X ratio	0.57	0.66	0.71

^1^Wheat millrun

^2^Barley brewers grain

^3^Macadamia nut cake

In vitro digestibility of starchy and fibrous feedstuffs is presented in Table 3. Among starchy feedstuffs, OSP had significantly higher (*P* < 0.001) in vitro ileal digestibility of DM (IVIDDM) than taro. Among fibrous feedstuffs, IVIDDM was significantly higher in MNC (*P* < 0.001) than BBG.

**Table 3:** Apparent in vitro ileal digestibility of nutrients of starchy and fibrous feedstuffs, %

Variables	OSP^1^	Yam	Taro	WMR^2^	BBG^3^	MNC^4^	SEM	*P*-value
Dry matter	80.1^a^	74.8^b^	63.5c	59.6^d^	49.1^f^	53.1^e^	0.006	<0.0001
Gross energy	84.2^a^	78.7^ab^	67.8^b^	62.3^bc^	53.1^d^	57.1^c^	0.004	<0.0001

^1^Okinawan sweet potato

^2^Wheat millrun

^3^Barley brewers grain

^4^Macadamia nut cake

^a,b,c^Mean values within a row with different superscript letters were significantly different ( *P* < 0.05)

**Fig. 1:**
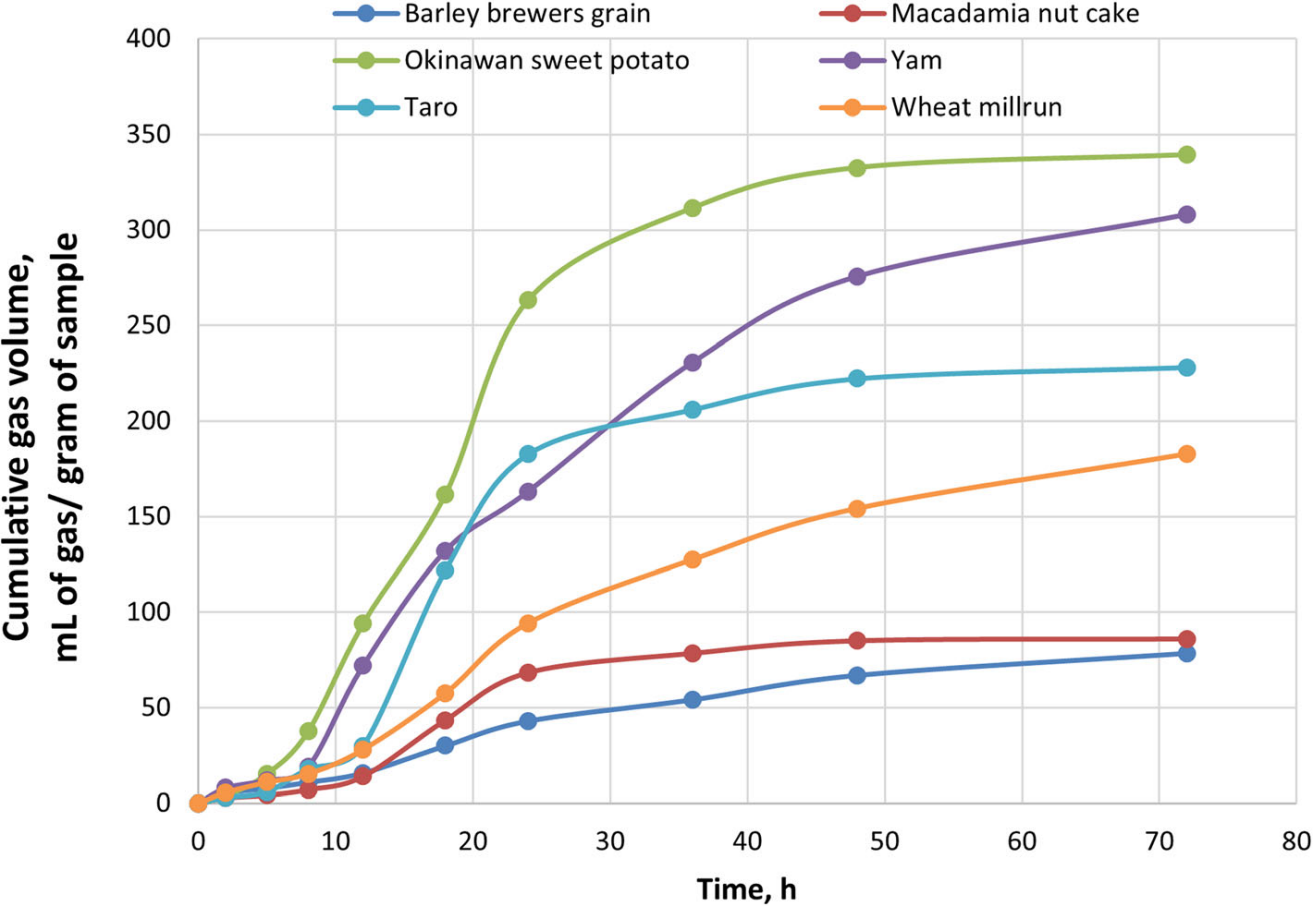
Cumulative gas production of starchy and fibrous feedstuffs in the large intestine of pig, studied using an in vitro model

The cumulative gas production among different feedstuffs varied widely (Fig. 1). In general, all the starchy feedstuffs fermented more extensively than fibrous feedstuffs. The OSP and yam have the highest cumulative gas production, while BBG and MNC had the lowest (*P* < 0.05).

Total SCFA production was significantly higher (*P* < 0.001) in OSP, taro, and yam than in BBG and MNC; WMR was in between (Table 4). Butyrate production was significantly higher (*P* < 0.001) in all starchy feedstuffs than MNC and BBG. Both acetate and propionate production was significantly higher (*P* < 0.001) in all starchy feedstuffs than BBG and MNC whereas, WMR was in between these feedstuffs. Thus, WMR fermentation behaved both like fiber and starch.

**Table 4:** In vitro fermentation metabolites of starchy and fibrous feedstuffs, mmol/g of sample incubated

Variable	OSP^1^	Yam	Taro	WMR^2^	BBG^3^	MNC^4^	SEM	*P*-value
Acetate	12.74^a^	13.12^a^	12.79^a^	10.29^ab^	7.57^c^	6.27^c^	1.197	<0.0001
Propionate	7.89^a^	7.59^a^	8.75^a^	5.21^ab^	3.71^b^	3.56^b^	0.832	<0.0001
Butyrate	3.19^a^	3.37^a^	2.92^a^	2.17^ab^	1.24^b^	0.97^b^	0.37	<0.0001
BCFA^5^	0.54	0.79	0.78	0.69	0.53	0.47	0.098	0.072
Total SCFA^6^	23.44^a^	24.87^a^	25.25^a^	18.36^b^	13.05^c^	11.27^c^	2.423	<0.0001

^1^Okinawan sweet potato

^2^Wheat millrun

^3^Barley brewers grain

^4^Macadamia nut cake

^5^Branched-chain fatty acid

^6^Short chain fatty acid

^a,b,c^Mean values within a row with different superscript letters were significantly different (*P* < 0.05)

**Fig. 2:**
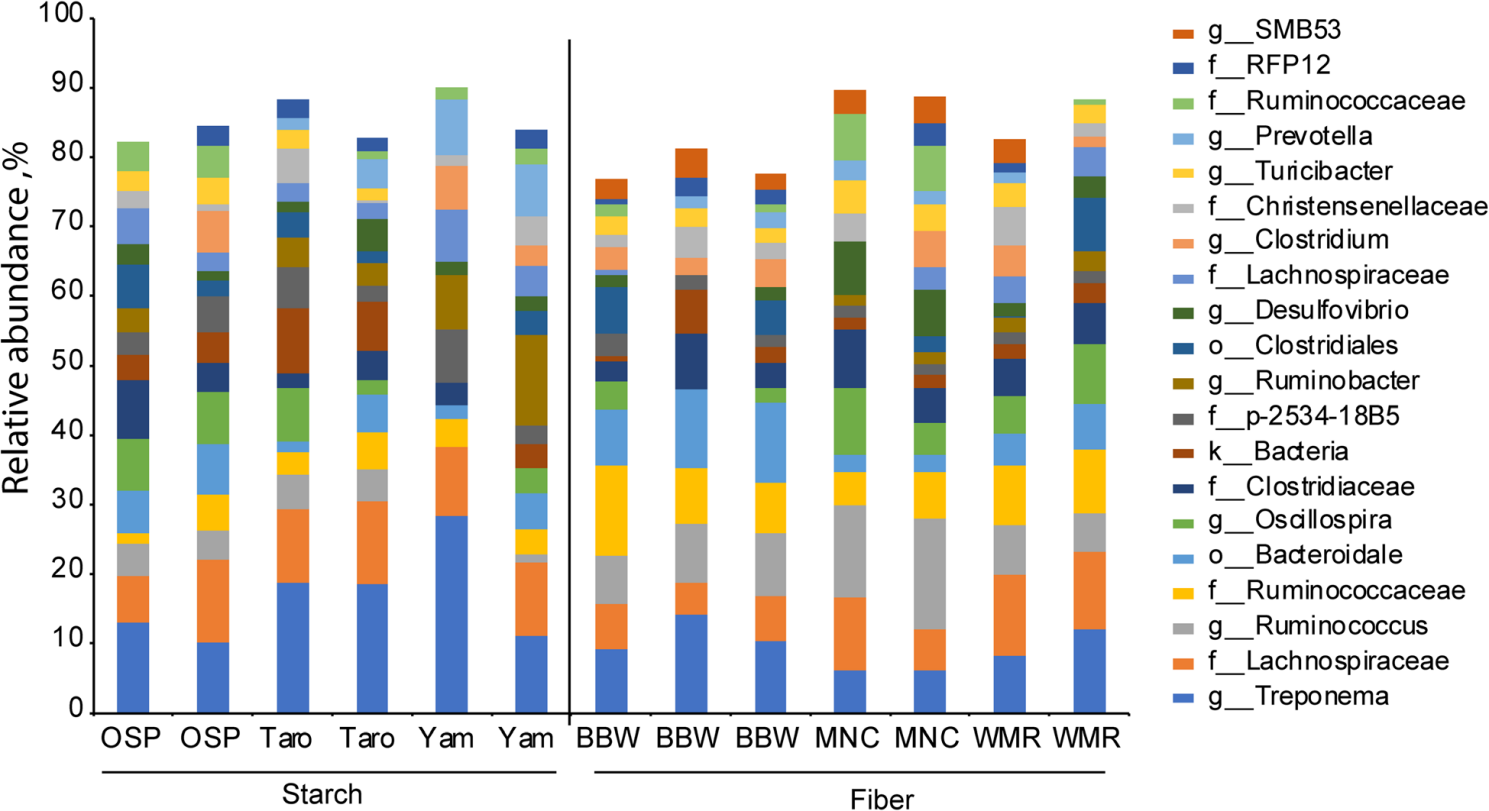
Relative abundance, % of microbiota in different starchy and fibrous feedstuffs in the large intestine of pig, studied using an in vitro model

**Fig. 3:**
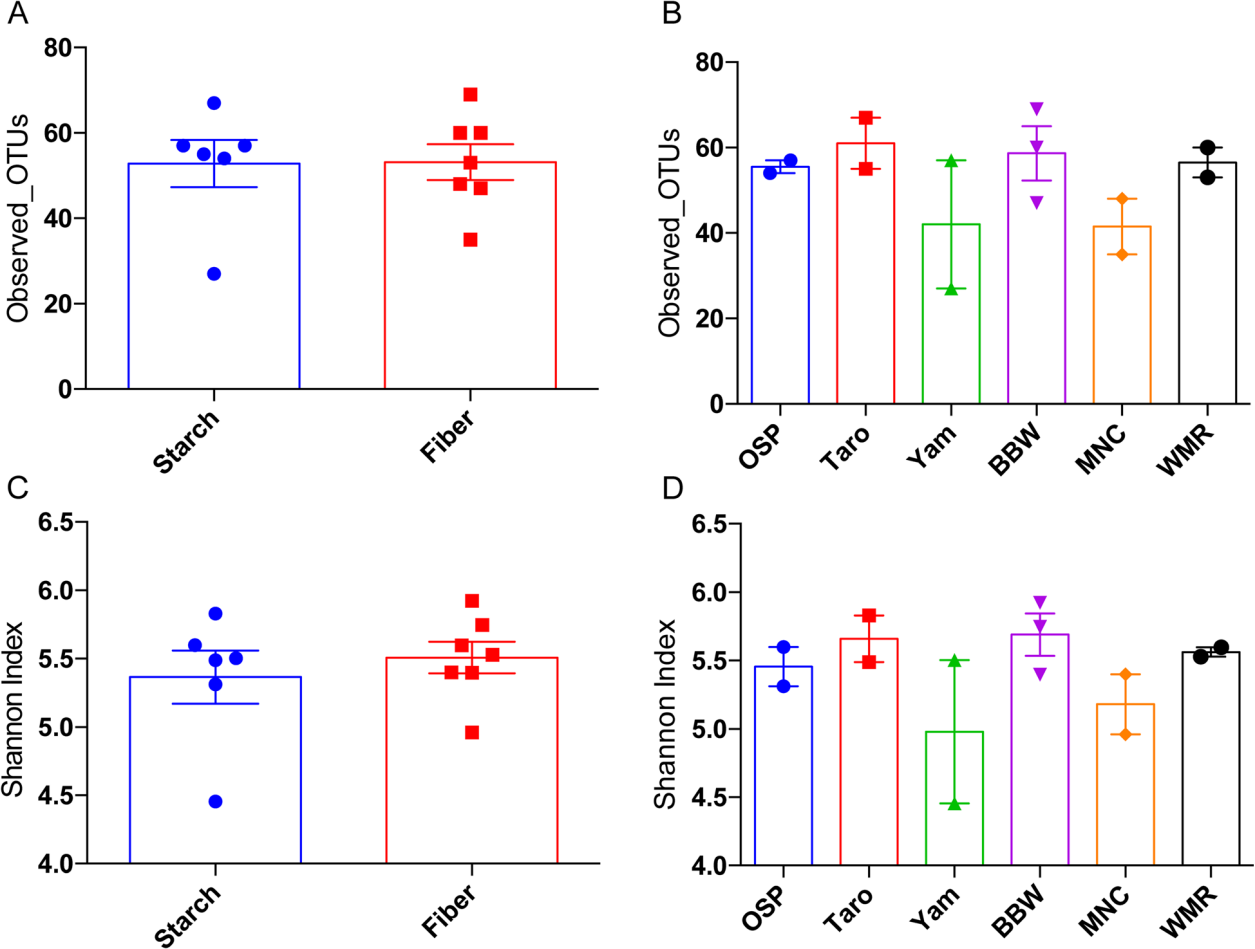
Alpha diversity of microbiota of different starchy and fibrous feedstuffs in the large intestine of pig, studied using an in vitro model

The taxonomic assignment at the genus level is shown in Fig. 2. The bar graph shows the relative abundance (%) of the top 20 genera in different feeds. Alpha diversity (bacterial composition within samples) of feeds is shown in Fig. 3. Alpha diversity of feeds at 10,000 reads per sample was measured with Observed OTUs (A and B) and Shannon index (C and D). Despite the difference between the types of fiber present in different fibrous feedstuffs tested, overall alpha diversity was not significantly different within and between starchy and fibrous feedstuffs. None of the groups were significantly different.

**Fig. 4:**
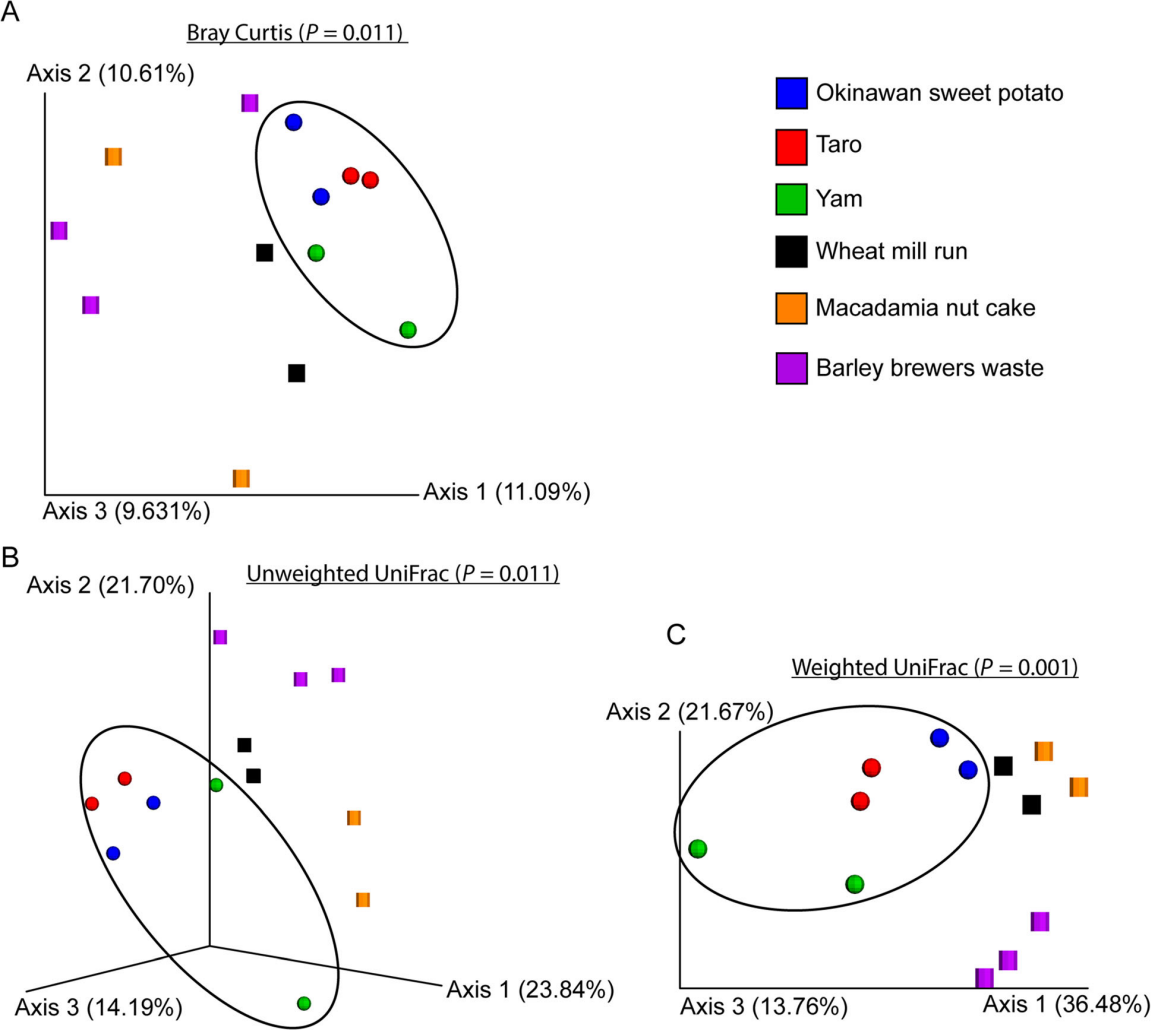
Beta diversity of microbiota of different starchy and fibrous feedstuffs in the large intestine of pig, studied using an in vitro model

**Fig. 5:**
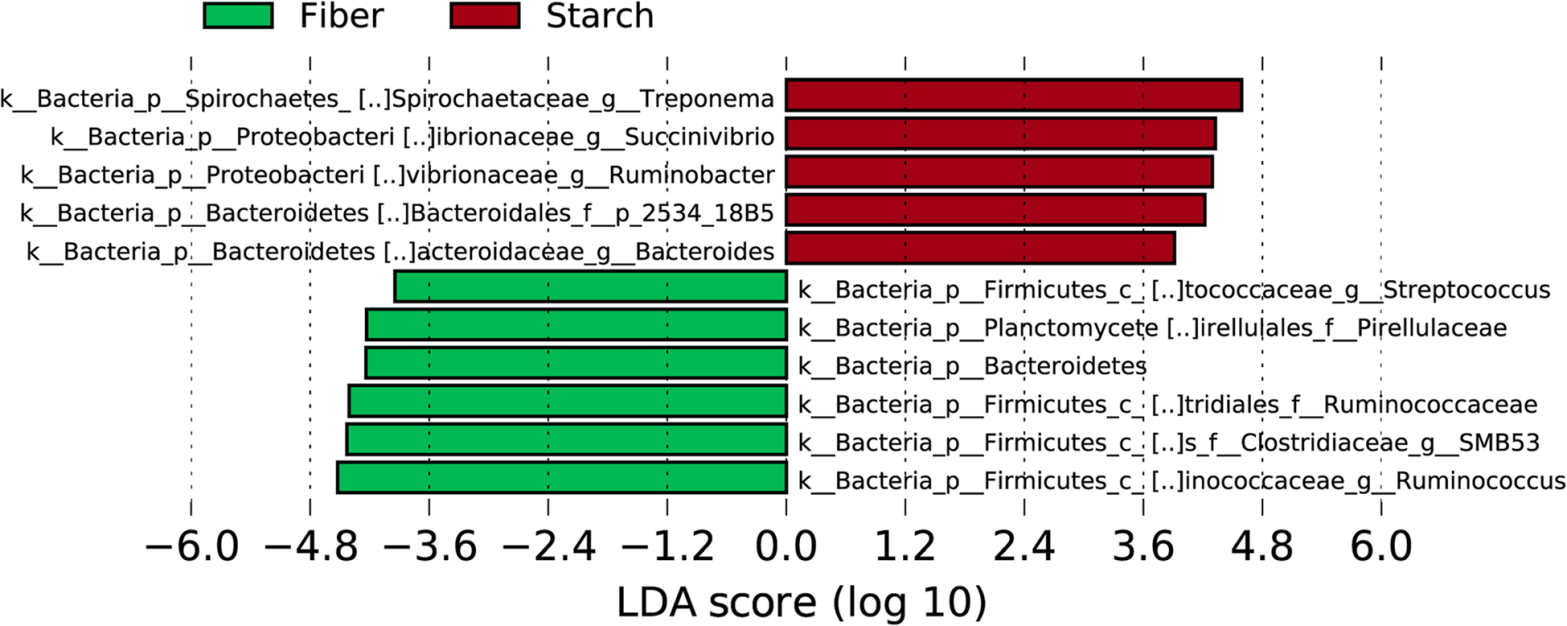
Linear discriminant analysis (LDA) effect size (LEfSe) analysis of microbiota (genus level) in different starchy and fibrous feedstuffs in the large intestine of pig, studied using an in vitro model

Beta diversity (bacterial composition between samples) of feeds is shown in Fig. 4. Beta diversity of feeds was performed at 10,000 reads per sample and measured using Bray Curtis dissimilarity distance (A), unweighted UniFrac distance (B), and weighted UniFrac distance (C). Starch-based feeds are encircled in Fig. 4. Beta diversity of starch and fiber feeds were significantly different from all three metrics (*P* < 0.05). The Fig. 5 indicates differentially abundant bacteria between fiber and starch-based feeds at the genus level.

## Discussion

Agricultural products and agro-industrial co-products can be used as partial substitutes of common energy ingredients in pig diets, especially for the subsistent farming system where agricultural products are not grown or are not widely available. These feedstuffs can also be used to replace traditional feed ingredients to some extent and can serve as a potential source of protein. These feedstuffs, however, contain high fiber or resistant starches and cannot directly be digested by the digestive system, but bacteria in the large intestine of swine can ferment them [1]. The gut microbiota composition is intimately connected to the host’s health and plays a crucial role in disease.

It is well known that the higher the lignin content, the lower is the fermentation. Thus, higher lignification in MNC can be the reason for its poor fermentation [9]. The higher amount of insoluble NSP in MNC and BBG can be another reason for its poor fermentation as insoluble NSPs are not easily and readily degraded [4, 9]. The higher amount of soluble NSP can be the reason for the higher amount of cumulative gas production and higher amount of SCFA production in WMR compared to another fibrous source. Soluble fiber is easily degraded by microbial enzymes and becomes available for microbial fermentation [14]. The fiber structure subjected to fermentation can influence the digestion and fermentation of feedstuffs [14]. Arabinose to xylose ratio (A:X) in MNC is comparatively higher than WMR and BBG, making MNC more difficult to degrade. Hence, a higher A:X ratio can be another reason for the poor fermentation of MNC. Starch and NSP ferments differently. However, starch which is heavily branched, or the one that contains a higher amount of amylopectin, provides a larger surface area for the enzymes to act on; hence gets broken down into smaller fragments (monomers and dimers) and are rapidly fermented. In contrast, the degradation of a more linear polymer or the RS containing a large amount of amylose and a low amount of amylopectin is slowly fermented [1]. Linear arabinoxylan and branched RS are rapidly fermented.

The composition of feedstuffs subjected to fermentation depends on the ileal digestibility, as all the undigested residue from the ileum is available for fermentation in the large intestine. The gas production indicates that all the starchy feedstuffs are fermented more extensively than fibrous feedstuffs. This suggests that RS in starchy feedstuffs though behave like fibers but is more extensively fermented than NSP present in the fibrous feedstuffs. The fractional rate of degradation of starchy feedstuffs is faster than the fibrous feedstuffs [1]. Fibers are degraded slowly as microbes take longer to break the larger polymer into smaller fragments, while starches are readily broken down and are rapidly fermented [15]. In other words, fermentation starts only after the substrate (arabinoxylan, β-glucan or RS) gets depolymerized by microbial hydrolytic enzymes. The faster the substrate’s depolymerization rate, the faster the carbohydrates will be available for fermentation by the bacteria [1]. Since the rate at which starch or resistant starch gets depolymerized is quicker than NSP, they are rapidly fermented in the proximal part, whereas arabinoxylan and other NSP get fermented slowly in the distal part of the large intestine [3]. Therefore, readily fermentable residues are present in the undigested residues of starchy feedstuffs than fibrous feedstuffs that were not digested by enzymes in the upper gut.

Among fibrous feedstuffs, WMR was more rapidly fermented than BBG and MNC. WMR behaved largely like starchy feedstuffs than fibers. The total gas production of WMR was closer to taro. The higher amount of soluble NSP in WMR can be the reason for the rapid and complete degradation [16]. MNC and BBG were alike, the fractional rate of degradation was slow, and the total amount of gas produced was much lower. It represents incomplete fiber degradation signifying the role of lignification, fiber structure (A:X ratio), and the amount of soluble vs. insoluble fiber. The higher amount of cumulative gas production indicates a larger amount of fiber degradation and a higher degree of fermentation [17]. Among starchy feedstuffs, OSP was rapidly fermented. The higher amount of fiber in taro can be the reason for the slow degradation of taro. OSP was rapidly and completely degraded, whereas taro was slowly fermented. When available, microbes preferably and readily ferment carbohydrates than protein [18]. Hence, saccharolytic fermentation occurs predominantly in the proximal colon and proteolytic in the distal colon as a less amount of carbohydrates is available [2]. Fermentation in the distal part of the intestine is desirable, and taro (being starchy still fibrous) and WMR can play a better role in lowering protein fermentation as it starts to degrade slowly as its fractional rate of degradation is slow and fermentation peaks up in the latter half. However, all the fibrous feedstuffs start to ferment slowly, but BBG and MNC are not extensively fermented, as shown by a lower volume of cumulative gas production.

Starchy feedstuffs produced more butyrate than fibrous feedstuffs high in arabinoxylan like MNC. Production of butyrate due to fermentation of RS was found two times higher than that produced due to fermentation of other NSPs [19–21], which is in accordance with this study where butyrate production was not twice as high but higher than fibrous feedstuffs. The most abundant genera present due to starchy feedstuffs were *Bacteroides*, *Treponema*, *Succinivibrio*, *Ruminobacter*, whereas fibrous feedstuffs resulted in more abundant *Streptococcus*, *Ruminococcus*. There was a significant increase in the *Bacteroides* population when swine were supplemented with RS type 4 [22]. Thus, RS type 4 can be the reason for the higher increase in number of *Bacteroides* in starchy feedstuffs. The molar ratio of butyrate production is affected by the source as well as the amount of substrate (RS) available for the microbes for fermentation [23], which ultimately will influence the proliferation of butyrate-producing bacteria [24]. The energy provided by butyrate is vital to maintaining the gut ecosystem and the health of swine. In the absence of energy (butyrate), fermentation is shifted towards amino acids [15]. On the other hand, fibrous feedstuffs resulted in an abundance of *Ruminococcus*, which increased butyrate production. An increase in the proliferation of *Ruminococcus* is associated with increased butyrate production and improved gut health [25]. An increase in butyrate production not only results from increased accumulation of butyrate-producing bacteria but can result from increased acetate and lactic acid produced by Bifidobacterium as 90% of butyrate is derived from the acetate [26]. Acetate and lactic acids are produced due to fermentation by *Bifidobacterium*, and butyrate-producing bacteria can consume those acetates in the gut [27, 28]. The amount of acetate produced by all the starchy feedstuffs in this study was higher than that produced by fibrous feedstuffs. Higher acetate produced by *Bifidobacterium* would have been consumed by butyrate-producing bacteria resulting in higher production of butyrate in starchy feedstuffs, whereas lower acetate production resulted in lower production of butyrate in fibrous feedstuffs. However, the amount of acetate produced due to fermentation of WMR was closer to taro, and so was the butyrate production. Two enzymes (butyryl-CoA and acetate Co transferase) transfer CoA moiety from butyryl-CoA to external acetate, which results in the production of acetyl CoA and butyrate. In the absence of acetate, 75% of the supplied glucose is converted to lactate; however, the presence of acetate results in the butyrate production [29]. Starchy feedstuffs increased the abundance of Bacteroidetes phylum, whereas fibrous feedstuffs mainly increased the proliferation of bacteria from phylum Firmicutes. Most of the studies done either in vitro [30], in vivo [31], or with humans [32] reported that butyrate production is relatively higher due to the fermentation of RS than of NSP. A similar pattern of butyrate production was observed in this study as well.

Starchy feedstuffs resulted in more BCFA production than fibrous feedstuffs. This indicates that starch got rapidly and readily fermented in the proximal part of the large intestine. The distal part, where carbohydrate was minimal, resulted in more protein fermentation, producing more BCFA. On the other hand, fibrous feedstuffs resulted in lower BCFA production, indicating a lower protein fermentation [14].

Both starchy and fibrous feedstuffs had no significant impacts on the alpha diversity. However, the beta diversity of starchy feedstuffs differed from fibrous feedstuffs indicating starch and fiber may influence species replacement (change in species taxa) and species sorting (change in abundance). Fibrous feedstuffs especially were able to shape the community structure compared to the starchy group. This might be because different microbes prefer different fiber types. *Bacteroides* prefer larger polysaccharides, whereas *Bifidobacteria* prefer oligosaccharides over monosaccharides and polysaccharides [1]. Though other starchy feedstuffs produced a variable amount of SCFA, their impact on the beta diversity of microbiota was minimal. They clustered together, indicating that they helped the proliferation of similar microbes. This might be because of the similarity in the type of RS present in all three starchy feedstuffs tested in the study. Microbial diversity of fibrous feedstuffs, on the other hand, were found scattered. Structural heterogeneity (indicated by A:X ratio), physiochemical properties of fiber (solubility), and concentration of different types of NSP (cellulose, mannan, AX) played a vital role and acted as a substrate for different types of microbes. The overall influence of fibrous feedstuffs in changing microbial ecology is higher compared to starchy feedstuffs.

## Conclusion

Starchy and fibrous feedstuffs ferment in a different pattern in an in vitro model of the porcine gut. Overall differences in the fermentation characteristics within and between starchy and fibrous feedstuffs can be attributed to their differences in NSP fractions and solubility. Feedstuffs rich in starch get rapidly and extensively fermented whereas, fibrous feedstuffs are slowly fermented. Starchy feedstuffs are potent butyrogenic substrates than fibrous feedstuffs. Starchy feedstuffs produce a large amount of SCFA than fibrous feedstuffs. Starchy feedstuffs clustered together, indicating they acted as a substrate for a similar type of microbes. On the other hand, fibrous feedstuffs having different concentrations of NSP and varying degrees of solubility can act as a substrate for the diverse microbial population signifying the role of diverse types and structural heterogeneity of fiber present in different ingredients.

## Data Availability

Not applicable.

## Abbreviations

BBG: Barley brewers grain
BCFA: Branched-chain fatty acids
GC: Gas chromatography
GIT: Gastrointestinal tract
IVIDDM: In vitro ileal digestibility of dry matter
MNC: Macadamia nut cake
NSP: Nonstarch polysaccharide
OSP: Okinawan sweet potato
RS: Resistant starch
SCFA: Short-chain fatty acid
WMR: Wheat millrun
